# Individual vices and institutional failings as drivers of vulnerabilisation

**DOI:** 10.1080/02691728.2024.2400083

**Published:** 2024-10-21

**Authors:** Havi Carel, Ian James Kidd

**Keywords:** illness, vices, mortality, vulnerability, vulnerabilisation

## Abstract

This paper explores the phenomenon of vulnerabilisation in relation to the experiences of persons with chronic illnesses. We distinguish a range of kinds of vulnerability, including epistemic vulnerabilities related to epistemic injustices, and describe various interpersonal and institutional processes which can create, exacerbate, and intensify those vulnerabilities. The dynamics of vulnerablisation are related to individual vices and institutional failings, the the pervasive pathophobia of many societies, and various contingent life-events. We conclude that susceptibility to varieties of vulnerabilisation is ultimately reflective of the fragility that is definitive of the human condition.

## Introduction

In this paper we explain the concept of *vulnerabilisation* and distinguish several ways individuals can be made vulnerable by interpersonal encounters and interactions with social structures. Specifically, we make these claims:

1. The concept of *vulnerabilisation* can help us better understand the experiences of persons with chronic illnesses.
2. We should attend to the *dynamics of vulnerabilisation*, including the ways it is enacted by individuals or by institutions and social structures.
3. The concepts of *individual vices* and *institutional failings* provide an explanatory framework to explore those dynamics.
4. Contingent external events play a significant role in vulnerabilisation and invite a shift in our understanding of life events, personal choice, and dependence.
5. Susceptibility to vulnerabilisation gestures to deeper facts about the human condition that can be articulated by concepts including contingency, dependence, and fragility.

This paper argues that the concept of vulnerabilisation can usefully connect three areas: philosophical analyses of illness, discussions about oppression and social justice and social epistemology. Vulnerabilisation is also a common feature of seemingly disparate groups – including ill persons, the disabled, those with learning disabilities, people who have suffered adverse childhood experiences, people deprived of education, refugees, people who experience frequent placement moves, neurodivergent persons, people living in poverty and deprivation, those who are elderly and frail and those traumatised by political or economic circumstances. This partial list shows that vulnerabilisation is complex, its sources varied and its effects on one’s life far-ranging. Moreover, since the causes and forms of vulnerabilisation are many and complicated, we need a nuanced taxonomy. Consider, for instance, that vulnerablisation can take different forms, such as creating, intensifying, exploiting, threatening, or making salient one’s vulnerabilities. A physical injury can create *vulnerability*, but that is different from *vulnerabilising* actions that then make that injury worse through neglect, for example, or actions that threaten or belittle the injured person.

People belonging to these groups can be subject to *vulnerabilising* forces, especially within institutions. We claim that these disparate groups share a susceptibility to vulnerabilising forces, especially within health and social care institutions, but also in education, criminal justice, employment, and other areas. In this paper we develop an understanding of what vulnerabilisation is and how it can be fuelled by processes that involve *individual vices* and *institutional failings*. In the background of these claims is a broader view of the human condition, which emphasises the role of contingent events in shaping one’s life, making us all hostage to fortune and more vulnerable than we presume or want to admit. Susceptibility to vulnerabilisation reflects a deep fragility that is, we argue, characteristic of the human condition across its diverse and contingent forms.

The paper proceeds as follows. Section one presents vulnerabilisation. Section two sets it within the ‘facts of life’ context developed in earlier work (Carel and Kidd 2019). Section three tracks one source of vulnerabilisation: individual failings. It then offers a vice-based analysis of these failings. A cluster of *pathophobic* vices are identified as driving much vulnerabilisation in the case of illness. Section four points to a further cause of vulnerabilisation, offering the concepts of *institutional failings* and *institutional opacity* to denote institutions that exacerbate and enable vulnerabilisation. Section five synthesises our understanding of vulnerabilisation as generated on both the individual and the institutional level.

### 1 What is vulnerabilisation?

Vulnerabilisation refers to processes, whether interpersonal, institutional, or socio-structural, that tend to cause individuals or groups to be vulnerable in a range of ways. ‘Cause to be vulnerable’ can mean different things – different *modes of vulnerablisation*, as we call them – which can include causing, exacerbating, intensifying, prolonging, or exploiting a person’s vulnerabilities. As a person is vulnerabilised, they are like likely to suffer a variety of harms, and so their situation worsens.

Vulnerabilisation has different causes, ranging from bad luck to mistreatment by others, to bad decisions on their part or that of others, through to the vicissitudes of life, such as injury, accidents, unavoidable misfortunes, and basic aspects of human life, such as the physical and cognitive decline that is integral to embodied life. Consider, for instance, the ways people are made *physically vulnerable* by injuries and diseases, in ways that can also feed economic, intellectual, and social vulnerabilities. Such persons can then be further vulnerabilised by sub-standard medical care, lack of decent housing, inadequate state welfare support and social neglect. Another example: people can be made *psychologically vulnerable* by trauma, harsh treatment, bullying, harassment, loneliness, and abuse. These can then be extended or exacerbated by lack of mental healthcare and support, emotional neglect, and untreated trauma.

A further example: *socially*, people are made vulnerable through exposure to violence and aggression, racism, classism, sexism, marginalisation, dehumanisation, systemic obstacles, and the discriminative and unfair realities integral to hostile social environments. Such people can then be vulnerablised if their identities are subjected to sustained assault by other people, social practices, or aspects of wider culture.

People are also *epistemically* vulnerabilised by being the targets of epistemically unjust treatment, epistemic exclusion, and epistemic violence, suffering the denial or depletion of testimonial credibility, having vital epistemic support withdrawn, being cast into hermeneutical lacuna, or being excluded from common epistemic practices (Dotson 2011; Fricker 2007; Hookway 2010). Different kinds of vulnerabilisation can interact, entrenching and intensifying one another. If all this is socially denied, even as it is radically institutionalised, these realities of vulnerabilisation will be concealed. A stark recent example is the UK’s now ex-Home Secretary, Suella Braverman, describing homelessness as ‘a lifestyle choice’.^1^ Put pessimistically, the scope, scale and severity of these and other forms of vulnerabilisation might be far greater than we usually realise (Benatar 2017). There are other ways in which persons can be vulnerabilised, too; this is not intended as an exhaustive list. Certain groups will suffer, for instance, political vulnerablisation, if their rights and entitlement to political participation are eroded or denied, while there may also be kinds of spiritual vulnerablisation.

Before moving on, we want to articulate several important aspects of vulnerabilisation. First, vulnerabilisation may be most harmful and most impactful at particular life-stages. Children and infants are disproportionately affected by, and vulnerable to, abuse; the effects of mistreatment can last a lifetime (Kirkengen 2010). Late life stages often entail loss or reduction of physical and cognitive capacities and diminished social confidence and connection which increase one’s susceptibility to vulnerabilisation (Bavidge 2017, Hughes 2023, Jaul and Barron 2017).

Second, the ubiquity of vulnerability should not reduce the need for its articulation, not least since there are a variety of obstacles to communicating its realities, ranging from ‘bright siding’ cultures to the ideologically motivated abuse and suppression of vulnerablised groups to more general patterns of wilful ignorance, indifference, and coldness (Ehrenreich 2009). Third, careful and systematic empirical research and concrete case studies are needed to advance our understanding of such processes (for one pioneering case study, see Tremain 2021). Finally, the fact that humans, and perhaps most living organisms, are intrinsically vulnerable to certain kinds of processes—such as disease, injury, and death—does not mean we should not strive to remove, minimise, or mitigate the contingent vulnerabilisation of human life.

We all die; that is one of our vulnerabilities. But how we die remains a contingent question settled differently by contingent circumstance. We cannot eliminate death, but we can certainly strive to promote better ways of dying (Binnie 2017, 2019; Kehl 2006). Feminist theories of vulnerability distinguish between *intrinsic* and *contingent* kinds of vulnerability, the latter being those ‘caused or exacerbated by the personal, social, political, economic, or environmental situations of individuals or social groups’ (Mackenzie, Rogers, and Dodds 2014: 7). To take one example, cognitive, physical, mnemonic, or sensory deterioration in ageing may be unavoidable, but ageist biases, stereotyping and discrimination are, hopefully, not (Langman 2023; Pritchard-Jones 2017). Vulnerability may be shared, but it is not equally distributed. There are important, overt, and morally salient patterns in the forms, kinds, force, intensity, scope, duration, and consequences of vulnerabilities in the human world.

We want to distinguish the two terms – ‘vulnerability’ and ‘vulnerabilisation’ – to indicate two different aspects of our shared fragility. We are all vulnerable in the ways described above. But that vulnerability can be exacerbated and amplified by contingent factors, including the actions and omissions of others and sociopolitical and economic circumstance. We use the term ‘vulnerabilised’ following Shelley Tremain’s (2021) important observation, to point to the fact that many individuals are *made to be and to remain vulnerable* by the actions, omissions, and decisions of other people, as well as by material, political, economic, medical, educational, and legal circumstances. It is those aspects of vulnerability that can be addressed through social action which are the focus of this paper. Although there isn’t always a clear way to say where the vulnerability shared by all ends and vulnerabilisation begins, we focus here on human actions that *cause* vulnerabilisation or, in other words, the contingent suffering that sits atop of the vulnerability we all share.

For Tremain, vulnerability is neither natural nor intrinsic. It is not ‘a prediscursive inherent human trait’ but, rather, ‘a contextually specific social phenomenon whose politically potent and artifactual character’ ought to be ‘recognized and acknowledged’ (Tremain 2021: n.p). Tremain draws on Foucault’s concept of *eventalisation*, ‘a breach of self-evidence that exposes the singularity of a given practice or state of affairs’, to induce an appreciation that ‘things are not as necessary as they seem’ (Tremain 2021).

We see in Tremain’s work an important contribution but suggest a different account which acknowledges both intrinsic and contingent sources of vulnerability and sees them as existing in a complex dynamic of interdependence. In what follows we provide examples of how intrinsic vulnerability becomes entangled with contingent (and hence, preventable) vulnerabilising forces. Our goal is to offer a philosophical account of both vulnerability and vulnerabilisation and of how they combine. We think this account can be critically applied to cases in which vulnerability is seen as unavoidable to expose its contingent elements. Such entanglement can falsely naturalise or essentialise vulnerabilisation, which is what we militate against. While we do not deny the universal nature of our vulnerability (see section two) we call for vigilance lest man-made, preventable, misery should be uncritically included in that account.

Vulnerabilisation can occur through individual actions, interpersonal interactions, interactions within social or institutional environments or a combination of all of these. Consider, for instance, some typical sorts of vulnerabilising individual-level interactions: making one’s vulnerability worse by neglecting or exacerbating it, threatening to exploit or worsen someone’s injuries, commenting disparagingly on one’s disabilities, suddenly and inexplicably bringing up someone’s chronic illness in a conversation on career progression, manipulating a person by intimating willingness to use their past trauma, voicing attitudes which imply that one holds derogatory views about the other, and so on. Such behaviours are well-described in work discrimination and oppression, though they include also microaggressions (Freeman and Stewart 2024). As that research emphasises, such behaviours needn’t be done consciously or maliciously, and there is no need to explain them in terms of morally invidious goals—a point we return to in our discussion of individual vices.

Tremain’s term ‘vulnerabilisation’ is also intended to emphasise the dynamic character of vulnerability. It is not a fixed, natural or immutable state. The dynamics of vulnerabilisation can be attributed to the following processes. First, subjecting someone to violent, discriminatory, or degrading behaviour can cause the targeted person to adopt behaviours that reveal or increase their own vulnerability. For example, if someone is successfully gaslighted in ways that erode their epistemic agency, they will reduce or altogether cease to try to express their point of view. If someone internalises ableist self-conceptions, their epistemic agency may be reduced, curtailed, or otherwise thwarted. So vulnerabilisation can become exacerbated, more pervasive, and more internalised through these and similar processes of undermining a person’s agency and epistemic efficacy.

Second, resisting vulnerabilising behaviours, whether deliberately and consciously or not, incurs a complicated set of physical, mental, emotional, practical, financial, and interpersonal costs. Struggling to be heard is costly and exhausting. It may require the vulnerabilised person to go to particular environments (Houses of Parliament, a hospital board meeting, a demonstration), something that is often difficult or impossible for many ill and disabled persons. One needs to remain resilient and courageous in the face of dismissive or overtly ableist reactions. One can be seen as ‘difficult’ and ‘defiant’, thus suffering further gaslighting, epistemic reduction, and epistemic injustice. Moreover, such resistant behaviours are prone to be interpreted pejoratively and in ways that are stereotype-confirming – one is ‘angry’, ‘challenging’, ‘a difficult patient’. Given this, we suggest that vulnerabilisation be seen as an iterative process that shapes and restricts the vulnerabilised person’s agency and capacity to resist the very behaviours that continue to harm them.

An example from education: consider a child who loses confidence to the point where their behaviour (e.g., truanting or leaving the classroom) is seen as a problem rather than a response to an environment the child finds overwhelming. Such a child may then go on to be labelled a ‘risk’ and eventually may be excluded from school and then sent to another school, to which they will arrive with their existing difficulties now exacerbated by their history of exclusion. This is one of the myriad processes of vulnerabilisation that end up creating lives that can be nasty, brutish and short, such as the life of a street drug user or a person institutionalised with a learning disability.

One may object that the concept of vulnerabilisation is too broad and heterogenous to be meaningful. However, we believe that these features of the concept are virtues. They point to ways of ascertaining similarities between different vulnerabilised persons and groups. Much of what we say about, say, ill persons can also apply to other groups, and being a member of one group is associated with suffering further vulnerabilities, such as poverty and being the target of discrimination. Broad and heterogenous concepts can help us explore the dense web of intricate intersectional connections between the lifeforms of different groups. We also suggest that – without erasing any of the deep differences between the heterogenous groups and persons who are vulnerabilised – there are general features and similar moral and social aims that unite them, similar to Fanon’s (2001) view that the ‘wretched of the earth’ are interconnected.

Another reason to use a broad, loose grouping is because of the dependence of many members of these groups on institutions such as health and social care. For such individuals, reliance on institutions may be more pronounced and fraught than for others. It is important to bear in mind that many interactions with institutions are not through choice. We attend Accident & Emergency when we suffer an accident; we attend court when we are summoned; we attend an appointment at the job centre to keep our benefits.

The term ‘vulnerabilisation’ also reveals significant roles for the actions of individuals, groups, institutions, and societies in causing vulnerabilisation, which is largely – but not exclusively – created and sustained socially. In other words, it is up to us whether individuals who are already vulnerable are then further vulnerabilised through neglectful, discriminatory, or malicious social arrangements. Vulnerabilisation is artificial, even if entrenched. Consider an example from housing: social care, the probation officer and prison service fail to communicate so that an already vulnerable person is released from prison with no suitable accommodation secured for them, further vulnerabilising them by forcing them onto the streets or into a hostel. When there are broad events impacting the whole of society (such as the Covid-19 pandemic), those who are already vulnerable will be the first to suffer and will also suffer due to many of the measures ostensibly implemented to protect them (Aho 2022a; Green 2021; Ratcliffe and Dolezal 2023).

Finally, it is important to note that we are not using the term ‘vulnerabilised’ as a moral excuse for a person’s behaviour. We focus here on how people are treated interpersonally and by institutions, the unique challenges they face and how they become vulnerabilised. We are not directly concerned here with the complex issue of moral conduct. Rather, our interest here is with how a person becomes vulnerabilised in the first place, within the context of power asymmetry, social and economic injustice, abuse, and exploitation.

### 2 The ‘facts of life’

Before we move on to describe the two levels (individual and institutional) on which vulnerabilisation operates, we want to fill out some of the background assumptions informing our account concerning (a) the human life cycle, (b) context sensitivity and (c) what we call the ‘facts of life’, which sees humans as vulnerable, dependent on others and susceptible to external events (Carel and Kidd 2019, see also Aho 2022b).

Starting with the *life-cycle view*, we set ourselves against a set of assumptions, common in philosophy, which take a physically and mentally healthy autonomous adult as their tacit template. By contrast, the life cycle view emphasises the cyclical form of a human life. In significant stages of life, we are helpless and depend on others. We depend on others entirely before birth and during infancy, and when our physical and mental capacities diminish, for example in old age, we become dependent once again. Crucially, the period in between these should not be assumed to be one of stable health, autonomy, and immunity to calamity. Within adulthood we remain vulnerable to accident, illness, trauma and other forms of adversity (Macintyre 1999; Mackenzie, Rogers and Dodds 2014; Carel 2018).

Second, *context sensitivity*. Within philosophy it is often the case that the tacitly assumed subject of discussion is an able-bodied adult, typically also male and white. ‘Special cases’ are then discussed, like those of women, trans persons, children, those who are disabled, people of colour and so on. This obscures the diversity and heterogeneity of human lives and establishes particular instances as typical, definitive, or ideal, with other forms both derivative and lesser. One way to counteract this is to acknowledge the variety of changing forms of human (and nonhuman) lives and the complex social contexts within which they try to understand and pursue their lives.^2^

A third assumption concerns what we call the *facts of life*, which are rooted in our being conditioned by a variety of contingencies—bodily, social, and other factors that can, and often do, constrain us in deep and disquieting ways. We are embodied and so susceptible to injury, illness, and ageing. We are social creatures who form communities and cultivate relationships with others which often render us vulnerable to forms of abuse, abandonment, disappointment, exploitation, and oppression. We are dependent on others for flourishing, wellbeing, and initiating projects that aim to give structure and meaning to our lives. But others can undermine our flourishing, and our projects can fail in ways we cannot mitigate. Such facts of life are, we suggest, the transcendental conditions of vulnerabilising processes and serve to anchor our analysis in a broad shared framework (see Carel and Kidd 2019; Carel 2021). Gathering these assumptions together, we can say that three of the deeply interrelated features of human life are *contingency, vulnerability* and *dependence*. They are not periodic features of the lives of certain unfortunate individuals but universal features of human life as such. With this account of shared vulnerability in place, we now turn to the sources of vulnerabilisation – the *individual* and the *institutional* – and their interface.

### 3 Individual failings: vices and pathophobia

Vices often contribute to the vulnerabilisation of others. In order to understand individual (or interpersonal) vulnerabilisation, we briefly describe such vices. In character ethics, vices are failings of individual character. On a broad view, vices are character traits or dispositions with perceptual, cognitive, affective, and behavioural components. Some typical vices, recognised across a range of moral traditions, include arrogance, cruelty, dishonesty, enviousness, and selfishness.

There are two main normative models that seek to explain the badness of vices. *Consequentialism* defines vices as traits that either (i) tend to cause a preponderance of bad effects or (ii) tend to fail to cause a preponderance of good effects. These are so-called *productive vices* and *passive vices*, respectively (Kidd 2022: 89-90). Productive vices include cruelty, since the cruel person typically performs cruel actions, while laziness is a passive vice. Heather Battaly refers to these as *effects-vices* (Battaly 2014: ch.2).

The second model is *motivationalism*. It defines vices as traits that (i) express or manifest the absence of positive values, desires, and other motivational states (*absence vices*), (ii) express or manifest the presence of bad values and motivational states (*presence vices*) or (iii) some combination of these. Epistemic insouciance is an absence-vice: the insouciant person lacks a proper concern for epistemic goods and standards, such as truth or evidential warrant (Cassam 2018). Malevolence is a presence-vice since the malevolent person is defined by desires to cause needless and unwanted harm (Baehr 2010).

We suggest that the badness of many vices includes their vulnerabilising powers. Vices can be motivated by a desire to vulnerabilise others, and the bad effects of vices can be the vulnerabilisation of others. Talk of vices and vicious agents always refers us to the social contexts in which vice-concepts are understood and deployed and the social identities of those vicious agents. There are gendered vices, for instance, such as docility and timidity, and the history of philosophy is full of cases where vice-concepts are corrupted by prejudices and negative stereotypes (Dillon 2003; Tanesini 2016).

Certain passive vices will drive and trigger kinds of vulnerabilisation; consider vices defined by failures of attention, care and helping. A vulnerable young person in danger of sexual exploitation is due to be visited by a lazy social worker. They do a home visit, but no one answers the door. They leave a card but do not attempt another visit. The visit is forgotten, and the young person remains at risk or harmed. A vulnerable young person has just been further vulnerabilised.

Certain productive vices – such as cruelty – involve actions that exacerbate or exploit the vulnerabilities of others. For example, a prison guard who uses their power to humiliate prisoners. Motivational vices defined by the absence or presence of appropriate motivations are also vulnerabilising. The British nurse Lucy Letby seems to have been motivated by desires to harm infants in her care. Other vices involve the absences of proper moral and epistemic desires and motivations. In care settings staff can fail to check their notes, lack concern for the dignity of those in their care or fail in moral and epistemic responsiveness to the vulnerable. We do not mean to generalise here, and of course one should be careful in making attributions of vices to individuals.

While the literature emphasises the many forms and causes of this injury, there is a common theme: in the imperfect social and material conditions of healthcare, it becomes ever more difficult to properly comport oneself according to the moral standards constitutive of healthcare practice. Many UK nurses, for instance, report being extremely and consistently stressed, overworked, and underpaid. Under these conditions, it is impossible to properly fulfil the moral standards and exercise the virtue integral to healthcare practice. So, if our virtuous actions and motivations fail, we need to identify the causes (Čartolovni et al. 2021; Mewborn et al. forthcoming).

Let us now turn to a specific subset of vices, which cluster around what Ian James Kidd (2019) dubbed *pathophobia*. A common theme of discourses about illness is the callous attitude that persons with chronic illness often encounter. A genre of ‘angry pathographies’ emerged in the late 1970s that spotlighted these kinds of mistreatment (Huntsaker Hawkins 1999: 11ff). Such texts offer powerful testimonies to the moral realities of being ill within a world hostile or indifferent to their predicaments. The *Cancer Journals* of Audre Lorde, for instance, speak of her ‘fury at the outside world’s viciousness, the stupid, brutal lack of consciousness or concern that passes for the way things are’ – the ‘arrogant blindness’ of many healthy people or the ‘cold and silent eyes’ of women who forsook sisterhood by shunning her (Lorde 1997: 24, 25).

The vices evoked in Lorde’s testimonies, and others like them, have many sources, including intersectional prejudices. However, they also reflect *pathogenic vulnerabilities*, a term used by feminist theorists for a vulnerability arising from one’s illness. Mackenzie, Rogers, and Dodds distinguish two kinds. *Primary pathogenic vulnerabilities* arise from ‘morally dysfunctional or abusive interpersonal and social relationships’, or systems of ‘sociopolitical oppression or injustice’. Such primary vulnerabilities can involve, *inter alia*, stigmatisation, the negative stereotyping of persons with chronic illnesses and institutional failure to support them. Physical and emotional abuse of ill persons, exclusion of the ill from the workplace or the systematic neglect of the needs of ill persons are examples. *Secondary pathogenic vulnerabilities* occur when a ‘response intended to ameliorate vulnerability’ has the ‘paradoxical effect of exacerbating existing vulnerabilities or generating new ones’. Cases include underregulated social care systems that expose people to abuse by their carers (see Mackenzie, Rogers, and Dodds 2014: 9).

To explain the connection of individual vices to the idea of pathogenic vulnerability, we use Kidd’s concept of pathophobia (Kidd 2019; Kidd and Carel 2021: §3). This term refers to morally objectionable attitudes, actions and patterns of behaviour which target and track persons with chronic somatic illnesses. It is intended as an analogue to other concepts, such as sanism (also known as mentalism) or ableism, but specific to those with chronic illnesses (Lavellee and Gagné-Julien forthcoming, Nario-Redmond 2020). Pathophobic attitudes and kinds of behaviours are diverse, shaped by the social identity of the ill person, the changing particularities of their illness and, in some cases, pejorative conceptions of the illness (the moralisation of ‘lifestyle diseases’, for instance, or sexually transmitted infections). Pathophobia is a pervasive feature of the social and epistemic predicament of many chronically ill people and a main source of their suffering (Kidd and Carel 2025).

Kidd argues that pathophobia can be a feature of individuals, institutions, and social structures, as well as conceptions of a good life. We focus here on individual-level pathophobia and, led by Lorde’s work, describe *pathophobic vices*. Persons with chronic illness report vicious treatment at the hands of others – of being *rudely* ignored, *cruelly* refused help, *insensitively* talked about, or having their testimonies treated *dismissively*. There are five clusters of pathophobic vices. The *aversion cluster* include failures of interpersonal interaction. The *banality cluster* include failures to desire, acquire, and appreciate the complexity and particularity of experiences of illness and the *callousness cluster* involves various failures of empathic concern which devolve into sub-clusters of *abandonment* and *abuse*. In the *insensitivity cluster* are failings concerned with failures to be sensitive to the personal, intimate, distressing nature of experiences of illness. The *untruthfulness cluster* includes various failures of truthfulness about the realities of illness (see Kidd 2019: §4). These various vices and failings are related to the basic conditions for human life, such as understanding and communicating with other people and authentically participating in meaningful activities within shared structures of intelligibility. Interpersonal relationships, understanding and the ability to make sense of one’s own life and experiences are essential to a human life.

The varieties of pathophobic vices can connect in many ways to our theme of vulnerabilisation. For example, banality cluster vices involve failures to seek an appropriately complex understanding of ways of being ill. Experiences of illness are often difficult to understand, make sense of and describe, for many reasons (Carel 2018a, 2016). Subjection to pathophobic banality from other people exacerbates these hermeneutical challenges (Carel and Kidd 2014). On Kidd’s account there are two aspects of banality in this sense. First: one can fail to comprehend and respond properly to the *particularity of illness experiences*—the fact that illness experiences are unique even if they share symptoms, treatment pathways, cultural scripts and so on (see Carel 2018a; Carel 2012). For many ill persons, it matters deeply that this particularity is recognised and taken seriously by others. Bland assurances about silver linings and time healing all often fail to connect with these particularities (Conway 2007).

A second aspect of pathophobic banality is the failure to appreciate the *complexity* of experiences of illness. A migraine is more than ‘a bad headache’. The breathlessness of a person with a life-limiting lung condition is not like the breathlessness a healthy person feels after they sprint for the bus (Carel 2018b). Illness experiences are dynamic and involve complicated changes to our experiences of the body, space, time, other people, and society. In the case of many chronic illnesses the world itself starts to feel alien. A sense of the world as *Unheimlich* – of no longer being ‘at home’ in the world – is integral to experiences of chronic illness and pain (see Carel 2018a; Svenaeus 2011). In these cases, one faces real hermeneutical difficulties, made worse by the banal reactions of others. As Kathlyn Conway poignantly explains:

[illness] is a world unto itself, and we who find ourselves there discover that the usual resources for coping are sorely tried. We long to hear from someone who speaks from within personal experience and describes what it is really like to have cancer, to lose a leg, to become blind, or to feel the mind spinning out of control […] We want to hear not clichés but an acknowledgement that illness is not simply an opportunity for personal growth but a soul-wrenching encounter with loss, limitation, and the reality of death. (Conway 2013: 2)

Obliviousness, superficiality, and triteness are some of the specific vices in the banality cluster. The upshot is the hermeneutical vulnerablisation of the ill person – their need to be able to understand their experiences is made even more difficult by the banality of others. It feeds an abiding uncertainty about what to do, how to act and to react, which precludes confident comportment. In acute cases this can reflect and intensify a loss of trust in the world (Groves et al. 2023; Ratcliffe 2021).

We used the concept of pathophobia to illustrate how individual vices relate to vulnerability. These pathophobic vices contribute to the vulnerabilisation of persons with chronic illnesses. Other non-pathophobic versions of these vices will vulnerabilise other persons and groups in different ways.

### 4 Institutional failings and institutional opacity

To complete our framework, we now move from individual-level interactions that are vulnerabilising to the institutional level, to identify how institutions vulnerabilise people through two mechanisms: institutional failings and institutional opacity. *Institutional failings* are the myriad ways in which institutions can fail individuals – through thoughtlessness, impossible financial constraints, confusion, bad management, poor incentive structures, inattentiveness, or downright callousness – in ways that vulnerabilise persons who use their services. We see institutional failings as the institution-level correlates of vices: they are ways in which institutions undermine, ignore, frustrate, reduce, silence, and fail vulnerable persons, exacerbating their vulnerability. Within some contexts the majority of a particular institution’s users are already vulnerable, e.g. social care. For such individual users further vulnerabilisation is all the riskier, and so we should vigilantly look out for vulnerabilising processes there.

Consider the case of a teenage pupil with undiagnosed foetal alcohol spectrum disorder (FASD), a neurodevelopmental disorder caused by *in utero* exposure to maternal alcohol consumption. FASD is strongly associated with anxiety, social deficits, executive dysfunction, and emotional lability. However, the condition is largely underdiagnosed, so the pupil may have no recognition of their needs or support. They may try, but fail, to access education in a normal classroom setting. Over time, their frustration will increase and display itself as disruptive conduct. The school may respond by sanctioning the pupil. The child will then feel rejected by the school and over time give up altogether on attending. They may truant and become a safety worry for the school or be permanently excluded. Even if the child has the formal support provided by the local authority’s special educational needs department and the formal reviews and consultations take place, these may have little effect. After a prolonged period of experiencing educational failure, such pupils’ chances of engaging successfully with education and leaving school with qualifications is markedly reduced. Hence the young person becomes vulnerabilised, so that they have not only the FASD-related deficits and challenges but also a cumulative history of friction with educational institutions, which itself carries a risk of unemployment, poverty, and social disengagement.

In this case it may be that no particular person is to blame. It may be that the school have tried their hardest, that a GP has seen the child but failed to provide the correct diagnosis because FASD is under-recognised within health care services. It may be that school provided support, but it wasn’t the right kind. What has vulnerabilised the young person are systems and institutions that are slow to respond, lack the correct knowledge and lack the resources to help. These are institutional failings, and their outcome is vulnerabilisation.

The second kind of institution-level vulnerabilising mechanisms is what we termed *institutional opacity* (Carel and Kidd 2021). It may be that the pupil’s parents tried their hardest to understand the relevant education legislation and local arrangements, such as education tribunals, education and health care plans, SEN protocols and the statutory obligations of schools. But there are reams of documents and mountains of information, the legislation is impossible to understand without legal expertise and perhaps they do not have a computer at home. Perhaps they have no tools with which to research their child’s needs. It may be that they cannot get the correct information and advice. They want to help their child but the institutional responses to their requests for help simply direct them to more webpages that are opaque to them, written in jargon they do not understand.

Institutional opacity characterises situations where the institution is increasingly resistant to epistemic assessment and understanding by their agents and, especially, their users. In these worrisome cases, opacity *impedes* epistemic agency. Here are some of the ways people describe institutional opacity:

“I don’t understand how they’re making these decisions.”

“Sorry, how did they get from those data to these policies—I don’t get it at all.”

“Do you know who we’d talk to if we want to find out what the evidence was for their deciding to do this?”

Institutional opacity imposes high epistemic demands on agents and users, requiring the acquisition and exercise of knowledge, understanding, confidence, epistemic skills and communicative competences needed to meet the institution’s demands and to be able to navigate it effectively (for the example of healthcare, see Kidd and Carel 2021). When an institution is opaque, it frustrates individual-level epistemic agency. Who should I speak to? What are the right terms to use for making requests? Am I only likely to be taken seriously if I use the right language—and if so, what is that language?

Institutional opacity can vulnerabilise in several ways. First, opacity can erode the institutional user’s epistemic confidence and with it their capacity to enact the appropriate styles of interpersonal presentation and epistemic performance. When we cannot see the rules, norms, or underlying conceptions of credibility clearly, it is hard to be confident when engaging in communicative exchange. Social and epistemic confidence involves knowing how to act, interact and communicate with others. Otherwise, one is unable to make epistemic contributions to institutional processes and requests.

Second, an opaque institution conceals the precise nature and operations of the relevant economies of credibility. Agents and users need to understand how credibility is defined so they can adjust their testimonial performances. This may include, for instance, adopting alternative expressive styles consistent with institutional norms. When institutional economies of credulity are opaque, agents and users often suffer. They may worry that their recurrent failure to give ‘the right answers’ or to properly fulfil the informational requests of institutions means there is a hidden set of standards at work.

A third way that institutional opacity can vulnerabilise involves a sense of solidarity and collective trust. When one experiences an institution as opaque, one sees that institution’s decisions as arbitrary, its practices obscure and its reasoning impenetrable. One therefore starts to feel alienated from the institution, since it is now a source of confusion and frustration that obstructs one’s efforts to acquire necessary goods, including things on which our wellbeing and life might depend (benefit payments, education, social care, healthcare, justice).

Institutional failings, and in particular institutional opacity, can result in tangible – and indeed the most severe – forms of suffering. Consider the case of Jodey Whiting, who took her life in 2017, two weeks after her benefit payments were stopped due to her missing a work capability assessment because she was hospitalised at the time.^3^ Philippa Day took her life in 2019 after her benefits were withdrawn and the Department of Work and Pensions (DWP) refused her request for a home visit (she could not leave her home). Her sister, Imogen Day, said that Philippa’s ‘poor mental health meant she was not able to handle the battle with the DWP for the reinstatement of her benefits.’ Philippa wrote that her dealings with DWP made her feel ‘trapped’ and ‘isolated from the world.’^4^ At least sixty-nine suicides and suicide attempts have been linked in the UK to stopped benefits between 2014 and 2020.^5^ Indeed, the failures of the UK social care systems became so apparent that they were investigated and condemned by the United Nations (Alson 2018).

As we use the terms, vices are features of individuals while failings are features of institutions. Both are vulnerabilising. Individuals can vulnerabilise and so can structures (Kidd and Carel 2021). Moreover, often individual, and institutional failings enmesh, for example, in the case of the Metropolitan Police, famously described as institutionally racist and misogynistic first by an inquiry and then a report 24 years later. The Macpherson Report (1999), which investigated the Met’s treatment of the Stephen Lawrence murder, concluded the Met police was racist in its norms, practices and internal culture. Alongside individual racist officers, the institution itself was racist. These institutional problems were not limited to racism, as the Casey (2023) review, 24 years later, shows, and could not be explained away as the individual failings of a few ‘bad apples’ in a blameless institution. In March 2023, Baroness Casey wrote:

[t]he Met preferred to pretend that their own perpetrators of unconscionable crimes were just ‘bad apples’, or not police officers at all. So throughout this review, I have asked myself time and again, if these crimes cannot prompt that self-reflection and reform, then what will it take? (Casey 2023, p.7)

Individual vices and institutional failings mutually support one another. One way this happens is by having a culture of weak adherence to institutional rules that in effect enables vulnerabilisation to take place unchecked. Another way involves lacking robust whistleblowing procedures. A third is by institutions failing to train, nurture and support the people working in them. A fourth is institutional lack of respect for users, allowing their employees to do the same, or institutions that promote unaccountability, for example, those that are far from the public eye (e.g. care homes abuses).

### 5 Institutional failings, individual vices, and vulnerability

In this final section we put our concepts of vulnerabilisation, individual vices and institutional failings and opacity to work in a case study of ill persons who, according to an abundant body of testimonies, are often rendered multiply vulnerable in their interactions with healthcare institutions. This includes specific experiences of epistemic injustice which target and track persons with chronic somatic illnesses (Carel and Kidd 2014; Kidd and Carel 2016b, 2018, 2021). Being chronically ill requires epistemic work that is often exacerbated by the interpersonal and institutional structures of modern healthcare systems. Hospitals, for instance, are complex, difficult to navigate geographically, epistemically, and socially, highly technical in their goals and practices, daunting and formal in their language and built around complex processes. Moreover, like other areas of the social world, healthcare institutions are often marred by pathophobia (Kidd 2019). Pathophobic attitudes and actions can be present within healthcare institutions, a point summarised in the chilling remark of one person with Guillain-Barré syndrome (which causes total paralysis) – ‘I was no longer afraid of the disease, but of the [healthcare] system’ (Baier and Zimmeth 1986: 100).

We think that pathophobic vices and institutional failings come together to generate and intensify the vulnerabilisation of ill persons. And this is against a background of vulnerability already amplified by illness. To start with, ailing bodies and their forms of suffering impact on one’s epistemic and interpersonal capacities; one may be less able to articulate one’s thoughts or demand they receive due uptake. One may lack the confidence and energy needed for physically and cognitively demanding interpersonal epistemic practices. One may be distressed, existentially distraught or struggling through experiences of grief and fear. An additional source of vulnerability are the effects of treatment, like ‘chemo brain’ or fatigue, especially when these are not properly accommodated or understood. All of these can disrupt or diminish the epistemic and communicative confidence and abilities of chronically ill persons in ways that fulfil the negative epistemic prejudices integral to many stereotypes about illness and so frustrate those persons. These processes occur in social and institutional cultures that are themselves influenced by pathophobic assumptions, values, norms, and conceptions of life (Carel 2016a; Kidd and Carel 2021).

Against this background of vulnerability comes vulnerabilisation. A source of vulnerabilisation are suboptimal features of healthcare institutions, including the generic ones mentioned earlier, like their being hierarchical, bureaucratic, and ordered around complex procedures and professional norms. These features are not vulnerabilising *per se* but require constant vigilance against allowing the systems’ demands to overshadow the ill person and their needs. Approaches such as patient-centred care, whole person care and holistic approaches are designed, in part, to guard against the institutional machinery overpowering the humanist focus on the ill person and their needs. These approaches have made substantial progress in our understanding of patiency, and epistemic injustice is a further such effort (Carel and Kidd 2014; Kidd and Carel 2019).

There are also conflicting goals, power differentials, internal compartmentalisation, strict reliance on rules that can be baffling to outsiders and use of distinctive idiolects and norms of conduct. These can be sources of confusion, distress, frustration, resentment, and practical and epistemic incapacitation. Appointments and treatment can be delayed. Departments and staff suffer what seem like continuous communication problems. Critical decisions are made without being properly explained or delivered in a blizzard of acronyms and jargon. The testimonies of patients and their advocates are filled with critical descriptions of the epistemically frustrating effects of these standard features of healthcare institutions (Carel and Kidd 2014; Kidd and Carel 2016b). These features can further vulnerabilise and epistemically disable ill persons.

One problem with these conditions – psychological, interpersonal, structural – is that they can encourage the exercise of pathophobic vices. The ill person becomes irascible and frustrated, provoking aversive behaviour from healthcare staff. They try to explain the rhythms and pulses of their pain only to find an unblinking health professional asking them to restate their existentially rich description in terms of a numerical pain scale. The ill person can become isolated and drained, signalling their vulnerability to cruel and abusive people. As their emotional sensitivities intensify, other people fail to adapt and speak to and about them in tactless ways, blankly delivering a dire prognosis on a busy ward with no privacy or support. Finally, the ill person finds that few speak the truth about these experiences nor want to hear such truths. Almost every exchange is glossily masked by relentlessly cheerful bright-siding talk of overcoming struggles and ‘getting better’. Even in this sketch, one sees the interplay of institutional conditions and interpersonal pathophobic behaviour that threaten to arise from a perfect storm of fear, time poverty, stress, and underdeveloped interpersonal skills.

We think that bureaucracy, complexity, hierarchy, jargon, stereotyping, pathophobia and other problems collectively obstruct the practical and epistemic agency of persons with chronic illness: elsewhere we call this the *predicament of patients* (Kidd and Carel 2021). It is a predicament because it is complex, all-encompassing, and replete with obstacles, threats, risks, and dangers. All of these can cause, intensify, or exacerbate vulnerabilisation in different ways. In the case of institutional opacity, for instance, strains are placed on one’s epistemic and communicative agency, but those strains are then obscured because the problems they create can be attributed to the individual failings of institutional users – to ‘problem patients’ who won’t learn rules or follow instructions. The reasoning here may be: ‘we have worked hard to make the institution transparent and accessible! Therefore, any problems must be down to insufficiencies on the part of users.’ The mistakes in that reasoning can in turn be concealed in ways consistent with pathophobic prejudices. Some people have difficulties processing information and applying it to novel situations—a failure to understand this is pathophobic *banality*. Some struggle to follow rules, like those with pathological demand avoidance—a failure to appreciate this entails *insensitivity* to people’s deficits and needs. Some are suspicious of institutions and their operatives, like health professionals, police officers or social workers – a failure to understand why is often rooted in a refusal to acknowledge truths about the problems inherent in healthcare, policing, and social care. In these cases, there are messily entangled relationships between institutional failings and individual vices.

A further aspect of this problem is a tendency to use the concept of vice to condemn those who have been vulnerabilised. Consider people who call out institutional opacity and are then labelled *disruptive* or those with rigid behaviour patterns (e.g. a person with autism) judged as *difficult*. Consider those with limited amounts of what is nowadays called ‘resilience’ – they are *angry* or *uncooperative*. In all these cases, a person is subjected to a double whammy of *vulnerabilisation* and *pathologisation* of their resistance. Consider, for instance, that epithets like ‘cooperative’ and ‘difficult’ are typically defined relative to the norms and demands of institutional procedures, rather than by sensitivity to the needs, capacities, and predicaments of their users (cf. Potter 2016).

There is also the converse tendency to overuse virtue concepts as part of hyperbolic discourses which ‘bright side’, romanticise or glamorise efforts to cope with vulnerability. A poignant theme of many illness narratives are reports of instances of cowardice, wilful denialism, egotism and selfishness (see, for instance, Middlebrook 1996). Glossy moral stories or simplistic tales of virtuous heroism should be rejected in a truthful exploration of the messily complicated realities of human efforts to cope with their own vulnerability.

When an ill person is vulnerabilised by an opaque institution that is resistant to epistemic assessment and understanding and then derogated as disruptive or difficult, it becomes even tougher to overcome opacity. When the cost of trying is likely to be derogatory pathologisation, things are even more difficult. Usually, we react to opaqueness by initiating epistemic practices like questioning, but in the cases we’ve described, this is liable to be seen as disruptive. After all, questioning can slow down medical decision-making and take up time no one can spare. Under such difficult material, social and psychological conditions, it might seem that vicious behaviour is almost inevitable, although that is too gloomy a conclusion. Another way to go about this is to rethink our conceptions of virtue and vice, of what they entail, how they arise, who is capable of them, the conditions under which people flourish and our capacity reliably to determine the viciousness or virtuousness of some pattern of behaviour (cf. Carel 2017; Dillon 2012; Tessman 2005).

## Conclusion

This paper developed the notion of vulnerabilisation and anchored it, causally and explanatorily, in two parallel and intertwining processes: individual and institutional failings. The paper set out to distinguish universal and intrinsic vulnerability from vulnerabilisation, explain what vulnerabilisation is, what may cause it, how it plays out and who it affects. Our social justice claim was that diverse and seemingly disparate groups are united under the umbrella term of vulnerabilisation. We further articulated the two vulnerabilising processes: the first, on the individual level, is powered by individual vices, and we provided an example of pathophobic vices and their vulnerabilising impact on ill persons. The second, on the institutional level, includes institutional failings and institutional opacity, the way that an institution makes itself epistemically inaccessible. In closing, we suggest that forces of contingency are at play in human lives; an appreciation of these forces is necessary for understanding vulnerabilisation as externally foisted upon a person rather than an outcome of their actions and choices.

## Footnotes

1 https://www.bbc.co.uk/news/uk-67321319 (accessed on 14.11.2023).

2 Recent examples of this more inclusive, non-hierarchical mode of thinking include Robert Chapman (2023) on neurodiversity; Ed Yong (2023) on the plurality of perception; and Birch et al (2021) on consciousness and sentience in nonhuman animals.

3 https://www.bbc.co.uk/news/uk-england-tees-57570295 (accessed 15 November 2023).

4 https://www.theguardian.com/uk-news/2021/jan/27/inquest-finds-mother-took-overdose-after-removal-of-disability-benefits (accessed 15 November 2023).

5 https://www.theguardian.com/society/2020/feb/07/dwp-benefit-related-suicide-numbers-not-true-figure-says-watchdog-nao (accessed 15 November 2023).
